# Investigating the potential impact of PCSK9-inhibitors on mood disorders using eQTL-based Mendelian randomization

**DOI:** 10.1371/journal.pone.0279381

**Published:** 2022-12-29

**Authors:** Alisha Aman, Eric A. W. Slob, Joey Ward, Breda Cullen, Nicholas Graham, Donald M. Lyall, Naveed Sattar, Rona J. Strawbridge

**Affiliations:** 1 School of Cardiovascular and Metabolic Health, University of Glasgow, Glasgow, United Kingdom; 2 School of Health and Wellbeing, University of Glasgow, Glasgow, United Kingdom; 3 Medical Research Council Biostatistics Unit, University of Cambridge, Cambridge, United Kingdom; 4 Department of Applied Economics, Erasmus School of Economics, Erasmus University Rotterdam, Rotterdam, The Netherlands; 5 Erasmus University Rotterdam Institute for Behaviour and Biology, Erasmus School of Economics, Rotterdam, The Netherlands; 6 Cardiovascular Medicine Unit, Department of Medicine Solna, Karolinska Institute, Stockholm, Sweden; Forest Research, UNITED KINGDOM

## Abstract

Prescription of PCSK9-inhibitors has increased in recent years but not much is known about its off-target effects. *PCSK9*-expression is evident in non-hepatic tissues, notably the brain, and genetic variation in the *PCSK9* locus has recently been shown to be associated with mood disorder-related traits. We investigated whether PCSK9 inhibition, proxied by a genetic reduction in expression of *PCSK9* mRNA, might have a causal adverse effect on mood disorder-related traits. We used genetic variants in the *PCSK9* locus associated with reduced *PCSK9* expression (eQTLs) in the European population from GTEx v8 and examined the effect on PCSK9 protein levels and three mood disorder-related traits (major depressive disorder, mood instability, and neuroticism), using summary statistics from the largest European ancestry genome-wide association studies. We conducted summary-based Mendelian randomization analyses to estimate the causal effects, and attempted replication using data from eQTLGen, Brain-eMETA, and the CAGE consortium. We found that genetically reduced *PCSK9* gene-expression levels were significantly associated with reduced PCSK9 protein levels but not with increased risk of mood disorder-related traits. Further investigation of nearby genes demonstrated that reduced *USP24* gene-expression levels was significantly associated with increased risk of mood instability (p-value range = 5.2x10^-5^–0.03), and neuroticism score (p-value range = 2.9x10^-5^–0.02), but not with PCSK9 protein levels. Our results suggest that genetic variation in this region acts on mood disorders through a PCSK9-independent pathway, and therefore PCSK9-inhibitors are unlikely to have an adverse impact on mood disorder-related traits.

## Introduction

Cardiovascular disease (CVD) is currently the leading cause of mortality in the world with 32% of global deaths attributed to it in 2019 [1]. Low-density lipoprotein cholesterol (LDL-C) is one of the key causal risk factors for CVD and a range of potent treatments such as statins reduce LDL-C level in the blood and lower CVD outcomes. A more recent LDL-C treatment are the PCSK9-inhibitors (PCSK9i), and also lower CVD outcomes. The *PCSK9* gene is located on chromosome 1 and encodes the proprotein convertase subtilisin/kexin type 9 (PCSK9) protein whose function is to target LDL-C receptors (LDLR) for degradation. Certain mutations in the *PCSK9* gene lead to excess removal of LDL-C receptors which in turn lowers the uptake of LDL-C into the cell and ultimately lead to higher levels of LDL-C in the blood. PCSK9i are usually monoclonal antibodies that block the excess PCSK9, preventing the degradation of LDL-C receptors, thereby lowering circulating LDL-C levels [2]. PCSK9i are prescribed for the treatment of familial hypercholesterolemia or in patients with hypercholesterolemia and atherosclerotic CVD. They are also used in conjunction with statins or other lipid-lowering medications for additional reduction of LDL-C in blood [3]. Recently, price reduction of PCSK9i has led to an increase in prescription amongst eligible patients [4]. However, there is little information on potential adverse drug reaction (ADR) or off-target effects of PCSK9i.

Despite the main role of PCSK9 in lipid metabolism being established primarily in the liver, the gene has detectable levels of expression in non-hepatic tissues, including the brain [5]. There is recent evidence of an association between *PCSK9*, both protein level [5] and genetic variation in the *PCSK9* locus [6], and mood disorders and related traits, including depressive symptoms and neuroticism. Mendelian randomization (MR) studies have provided evidence, using genetic risk scores of variants within the *PCSK9* locus, that this gene is associated with an increased risk of major depressive disorder (MDD) but not neuroticism [7]. *In vivo* experiments have also demonstrated that overexpression of LDLR (which is targeted for degradation by PCSK9), in mice brains led to neuroinflammatory responses, suggesting that inhibition of PCSK9 may also lead to neuroinflammation [8]. MR studies have also found LDL-C to be associated, albeit weakly, with MDD-related traits [9]. Moreover, mental illnesses including MDD have well-established comorbidity with CVD [10]. In contrast to medications like statins, which have over 40 years of clinical data for analyses of ADRs and off-target effects [11], clinical data on the long-term effects of PCSK9i do not yet exist, with the first PCSK9i being approved by the FDA only in 2015 [12]. In such instances, statistical methods like MR can estimate potential ADRs, or off-target effects using genetic data, akin to ‘natural randomised control trial’ [13].

Two-sample MR is a technique used to estimate the causal association of a risk factor (exposure) on a phenotype (outcome) using genetic variants as instrumental variables (IV), a proxy for the risk factor, using summary statistics for genetic variants associated with the exposure and outcome derived from the GWAS of non-overlapping samples [14, 15]. In contrast to measurements of PCSK9 protein levels, genetic variants are stable across the life-course and not influenced by ongoing disease processes, so are ideal for investigating causal associations. Therefore, to assess whether PCSK9i might have adverse causal effects on mood disorders, we used an extension of the two-sample MR [16, 17]. Specifically, we used genetic variants associated with reduced *PCSK9* gene-expression (expression quantitative trait loci or eQTLs) to proxy the effects of PCSK9i (exposure) on mood disorder-related traits, circulating LDL levels, and PCSK9 protein levels (outcomes). Genetic effects on the outcome traits were identified from the respective largest publicly available genome-wide association study (GWAS) summary statistics for these traits.

## Materials and methods

### Exposure data

eQTLs are genetic variants which have genotype-specific effects on levels of the gene’s expression [18]. The principle behind our analyses plan is that the alleles for an eQTL which reduce the expression level of the gene, leading to lesser gene product, can be used as a proxy for the effect of a drug that reduces the level of the same gene product. Hence, we can use gene-expression level as the exposure to find the causal association with the outcomes of interest. Only cis-eQTLs, which are eQTLs within 1Mb window of the gene and on the same chromosome [18], were used in this study as the instrumental variables (IVs) for the MR analyses.

Summary statistics for significant cis-eQTLs were extracted from GTEx v8 [19] (49 tissues, n = 70–838, eQTL p<5x10^-8^) in the discovery analyses, and eQTLGen consortium (whole blood, n = 31,684, eQTL p<1x10^-8^) [20], CAGE consortium (peripheral blood, n = 2,765, eQTL p<1x10^-8^) [21], and Brain-eMETA dataset (meta-analysed data for brain tissue, n = 1,194, eQTL p<1x10^-8^) [22] in replication analyses.

### Outcome data

GWAS summary statistics with the largest sample sizes in European ancestry individuals were extracted using the IEUGWAS R package [23]. Outcomes considered were mood instability (id: ukb-b-14180, n = 451,619), neuroticism score (id: ukb-b-4630, n = 374,323), MDD (id: ebi-a-GCST005903, n = 217,584) [23, 24]. For positive controls, we used LDL-C (id: ieu-b-110, n = 403,943) [23, 24], and circulating PCSK9 levels (n = 12,721) [25]. Information on the GWAS studies used is detailed in S1 Table. If no GWAS summary statistic was available for an eQTL, data from its proxy at r^2^ threshold of 0.8 was used wherever possible. The cis-eQTL and GWAS summary statistics were combined, harmonized to the *PCSK9* gene-expression reducing allele.

### Power calculation

We used mRnd web version (accessed 19/10/2022) [26] to calculate the power of our SMR analyses using data for tissues having significant *PCSK9* eQTL with the highest (n = 670, whole blood) and lowest (n = 175, cerebellar hemisphere) sample size in the GTEx cohort. Wherever reported, we used the observed estimate for the effect of *PCSK9* gene expression on neuroticism score, MDD, and LDL-C. Where these were not available, we used reported estimates of *PCSK9* genetic risk score on the above outcomes instead [7, 27]. No reported estimates were available for *PCSK9* expression on mood instability and hence we could not calculate the power for this outcome. For the standard deviation (SD) of gene expression GWAS in the GTEx cohort, we used the reported value of log SD 0.13 as modelled in the initial cohort analyses [28]. We assumed the type-I error rate (α) as 0.05. The parameters used for the power calculations are available in S2 Table.

### Summary-based Mendelian randomization (SMR) analyses

SMR version 1.03 software was used to investigate whether reduced *PCSK9* levels had a significant causal effect on mood disorder-related traits. The detailed information on the SMR method has been described previously [17, 29]. In brief, SMR is an extension of MR that integrates GWAS and eQTL summary statistics to test if *PCSK9* gene-expression levels were associated with an outcome. The heterogeneity in dependent instruments (HEIDI) test, described previously [17], is also performed to explore if the observed association is due to another causal variant in high LD with our instrument instead of true causality (P_HEIDI_<0.1). The following settings for SMR were used: P_eQTL_<5x10^-8^, MAF<0.1, LD pruning threshold of r^2^<0.2, and exclusion of eQTLs in very high LD (r^2^>0.9) or in low or no LD (r^2^<0.05) with the top associated eQTL for the HEIDI test. For the discovery analysis, we used *PCSK9* gene-expression levels in 49 different tissues from GTEx v8 as exposure and mood-related traits and positive controls as outcomes. Limitations of cis-eQTL analyses include the significantly lower sample size of an eQTL study compared with the sample size of the GWAS used and the single time-point of expression data. These concerns, combined with the evidence of *PCSK9* gene-expression and cis-eQTLs in multiple tissues, justified our decision to use data from all available tissues in GTEx v8. SMR multi-SNP analysis was also performed to obtain a weighted estimate and p-value for all independent cis-eQTLs per gene. The p-value threshold was adjusted using Bonferroni correction for multiple testing. Furthermore, to explore whether a neighbouring gene, rather than *PCSK9*, might be the effector gene through which genetic variants in the *PCSK9* locus act, we also performed SMR for genes within 1Mb of *PCSK9* using the same IVs as that of *PCSK9* gene-expression. We also performed the SMR analyses with *LDLR* gene-expression level as exposure to assess if any association of PCSK9i on mood-disorder traits could be due to lower PCSK9 levels translating into higher LDLR levels.

### Cross-tissue MR estimate

We combined the eQTLs in all available GTEx v8 tissues and calculated a weighted estimate, modified and adapted from previously described cross-tissue IV selection methods [30, 31]. The ‘significant variant-gene pairs’ data was extracted for *PCSK9* and *USP24* from the GTEx v8 portal (https://www.gtexportal.org/home/datasets, accessed 02/02/2022). For each gene, the eQTLs were combined irrespective of its tissue. Where a variant was an eQTL in multiple tissues, the summary statistics for the tissue with lowest p-value was taken forward. These cis-eQTLs were used as IVs for subsequent MR analyses. The cis-eQTLs were harmonised with GWAS summary statistics and LD-pruned (r^2^<0.2 as above) to select only independent instruments. For every gene-phenotype combination, the Wald ratio was calculated for each eQTL followed by meta-analysis, using inverse-variance weighted (IVW), MR-Egger, weighted-median, weighted-mode, and maximum likelihood methods using the R-package TwoSampleMR (version 0.5.6) [32]. The MR-Egger intercept, and Cochran’s Q tests were used to assess directional pleiotropy and heterogeneity between SNPs [32, 33]. Bonferroni multiple-testing correction with an α = 0.005 was done for the total number of traits (five) analysed per gene.

### Colocalization analyses

We performed a genetic colocalization analysis with mood disorder traits and genetically modulated gene-expression levels of *USP24* within and ±1Mb flanking the gene. We used coloc v5.1.0.1 R package, the method of which has been described previously [34]. In brief, coloc uses summary statistics for two traits to conduct a Bayesian test for colocalization in a genetic locus between the pairs of genetic associations from the two studies. It also assumes that there is a single causal variant driving the association for both traits, and calculates 5 posterior probabilities, viz. H_0_: no genetic association with either trait, H_1_: association with trait 1 but not trait 2, H_2_: association with trait 2 but not trait 1, H_3_: association with trait 1 and trait 2 but with two independent causal variants, H_4_: association with both trait 1 and trait 2 and they share the same causal variant. Summary statistics for neuroticism score and mood instability from the same GWASs as the MR analyses, and *USP24* eQTL summary statistics from GTEx for all tissues were used for the colocalization analyses. The default priors were used: p_1_ and p_2_ = 10^−4^, and p_12_ = 10^−5^.

### Ethical approval

As all data used in this study is publicly available summary statistics from anonymised individuals, no ethical approval was required.

## Results

### *PCSK9* gene-expression is unlikely to be associated with mood disorders

**Fig 1:**
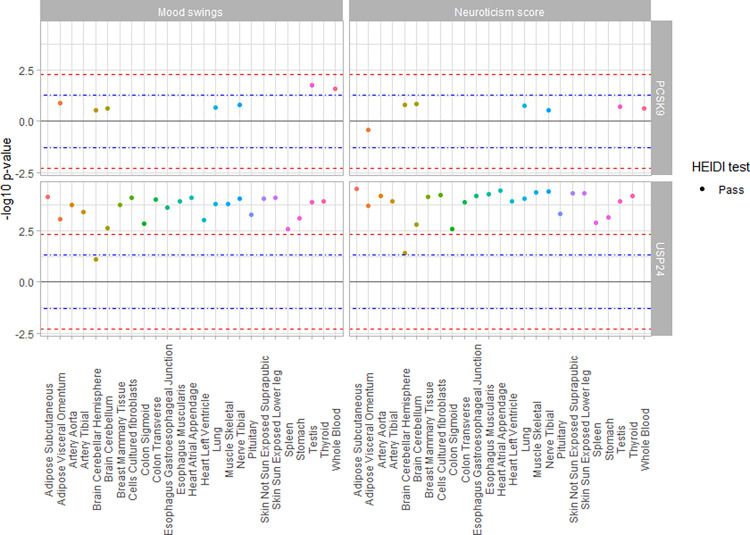
MR association of *PCSK9* and *USP24* gene-expression levels with phenotypes of interest. The Y-axis represents the -log_10_ p-value of the MR association, while the direction signifies the direction of effect of gene-expression reduction, which serves as a proxy for PCSK9i. SMR multi-SNP analyses association of the available 25 tissues for *PCSK9* and *USP24* gene-expression in GTEx v8 for outcomes Mood swings (A) and Neuroticism score (B). (C) MR (IVW) for *PCSK9* and *USP24* gene-expression with combined eQTL estimates from all available tissues in GTEx v8. The blue and red lines signify p-value of 0.05 and Bonferroni-corrected threshold for 5 traits per gene of 0.005, respectively.

SMR analysis was used to assess the association of *PCSK9* gene-expression with PCSK9 and LDL-C levels (as positive controls) as well as MDD, mood instability, and neuroticism score. The expected associations with *PCSK9*-expression and LDL-C levels were observed, and there was a suggestive association between *PCSK9*-expression and mood instability in testis (p_SMR_multi_ = 0.018, p_HEIDI_ = 0.07), and whole blood (p_SMR_multi_ = 0.02, p_HEIDI_ = 0.18), but this did not reach significance after multiple testing correction. No significant associations were observed between *PCSK9* gene-expression, and MDD, or neuroticism score (Fig 1 and S1 Fig, S3 Table). Our analyses were also sufficiently powered, mainly due to the use of outcome GWAS summary statistics from UK BioBank cohort, which has a very large sample size.

### *LDLR* gene-expression levels is unlikely to be associated with mood-disorder traits

To further assess if PCSK9i may be associated with mood disorder traits through the increase in LDLR levels instead, we conducted an SMR analysis using LDLR gene-expression levels as exposure and mood-disorder traits as outcome. We found that in the GTEx cohort, there was only one eQTL in whole blood that passed our significance filter and was not significantly associated with any of the mood disorder traits (S3 Table). We also replicated the analysis in eQTLGen consortium and find that increase in LDLR gene-expression was significantly associated with decrease in LDL-C levels, but was not associated with MDD, mood instability, and neuroticism score (S4 Table). We also found that change in *LDLR* gene-expression level is also not associated with change in PCSK9 levels, suggesting the absence of reverse causation.

### *USP24*, not *PCSK9*, is the effector gene for mood-related trait association

*PCSK9* shares some cis-eQTLs with nearby genes. To test whether another gene might be the effector gene on mood related traits, the analysis was repeated using the same instruments but their effects on expression of genes within ±1Mb of *PCSK9*. We found that the expression of the gene *USP24* showed suggestive evidence of association with mood instability (p_SMR_multi_ range = 0.01–0.04, p_HEIDI_ range = 0.35–0.55), and neuroticism score (p_SMR_multi_ range = 0.04–0.11, p_HEIDI_ range = 0.1–0.3) with the same instruments (shared eQTLs with *PCSK9*) (S1 Fig, S5 Table).

**Fig 2:**
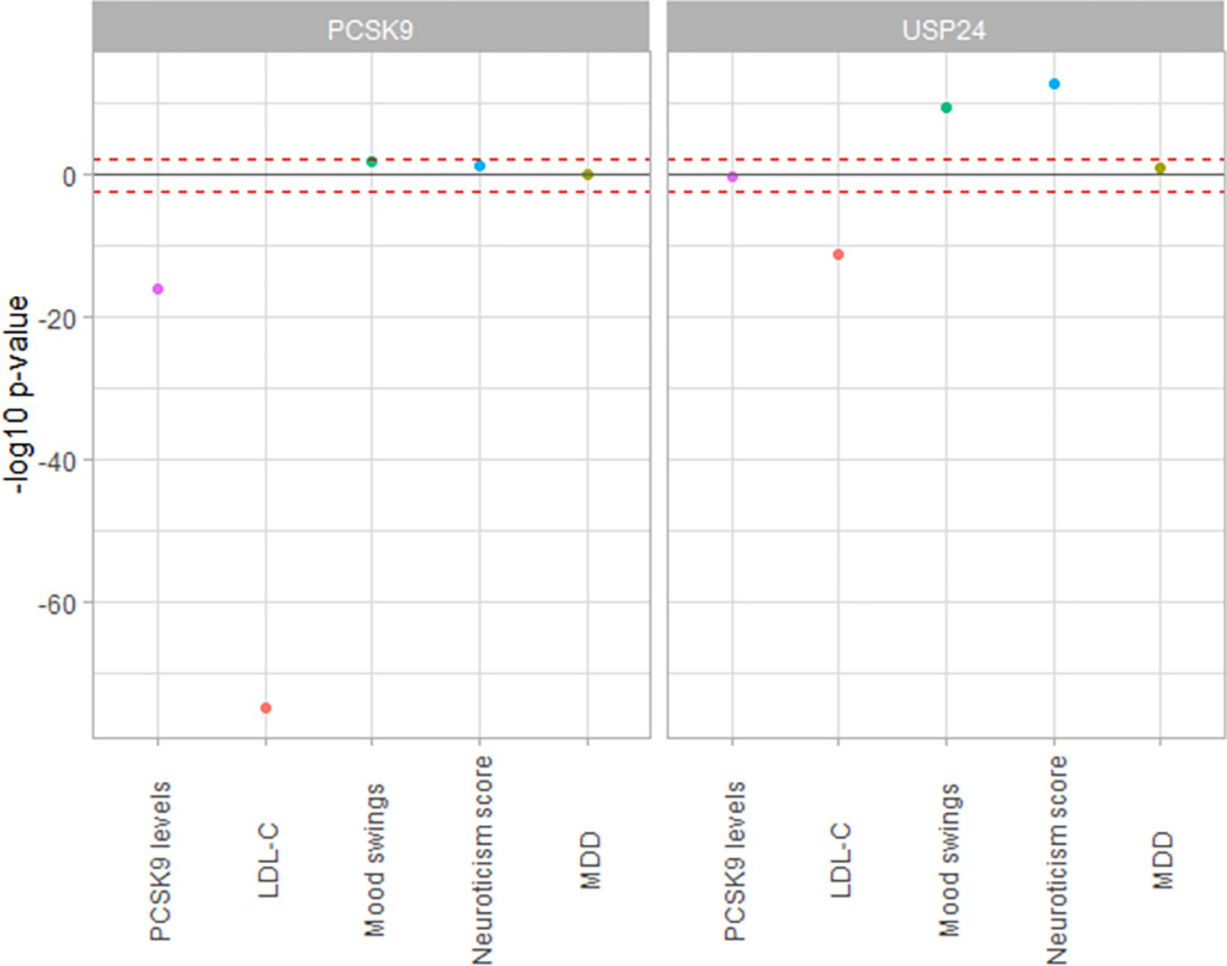
Cross-tissue MR association of *PCSK9* and *USP24* gene-expression levels with phenotypes of interest. The Y-axis represents the -log_10_ p-value of the MR association, while the direction signifies the direction of effect of gene-expression reduction, which serves as a proxy for PCSK9i. MR (IVW) for *PCSK9* and *USP24* gene-expression with combined eQTL estimates from all available tissues in GTEx v8. The blue and red lines signify p-value of 0.05 and Bonferroni-corrected threshold for 5 traits per gene of 0.005, respectively.

Expanding these analyses with all independent cis-eQTLs of *USP24* (those shared with *PCSK9*, and those specific to *USP24)*, we found a significant association for reduced *USP24* gene-expression with an increase in mood instability (p_SMR_multi_ range = 5.2x10^-5^–0.03, p_HEIDI_ range = 0.28–0.93), and neuroticism score (p_SMR_multi_ range = 2.9x10^-5^–0.02, p_HEIDI_ range = 0.18–0.99) in all tissues for which data was available (S3 Table). To further increase the confidence in our analyses, we performed a cross-tissue MR analysis by combining all its significant cis-eQTLs from all tissues into a single weighted estimate. We found no significant association for *PCSK9* gene-expression but significant association for reduced *USP24* gene-expression with an increase in mood instability, and neuroticism score (Fig 2, S6 Table).

### Replication of the effects of *USP24* on mood traits in additional datasets

**Fig 3:**
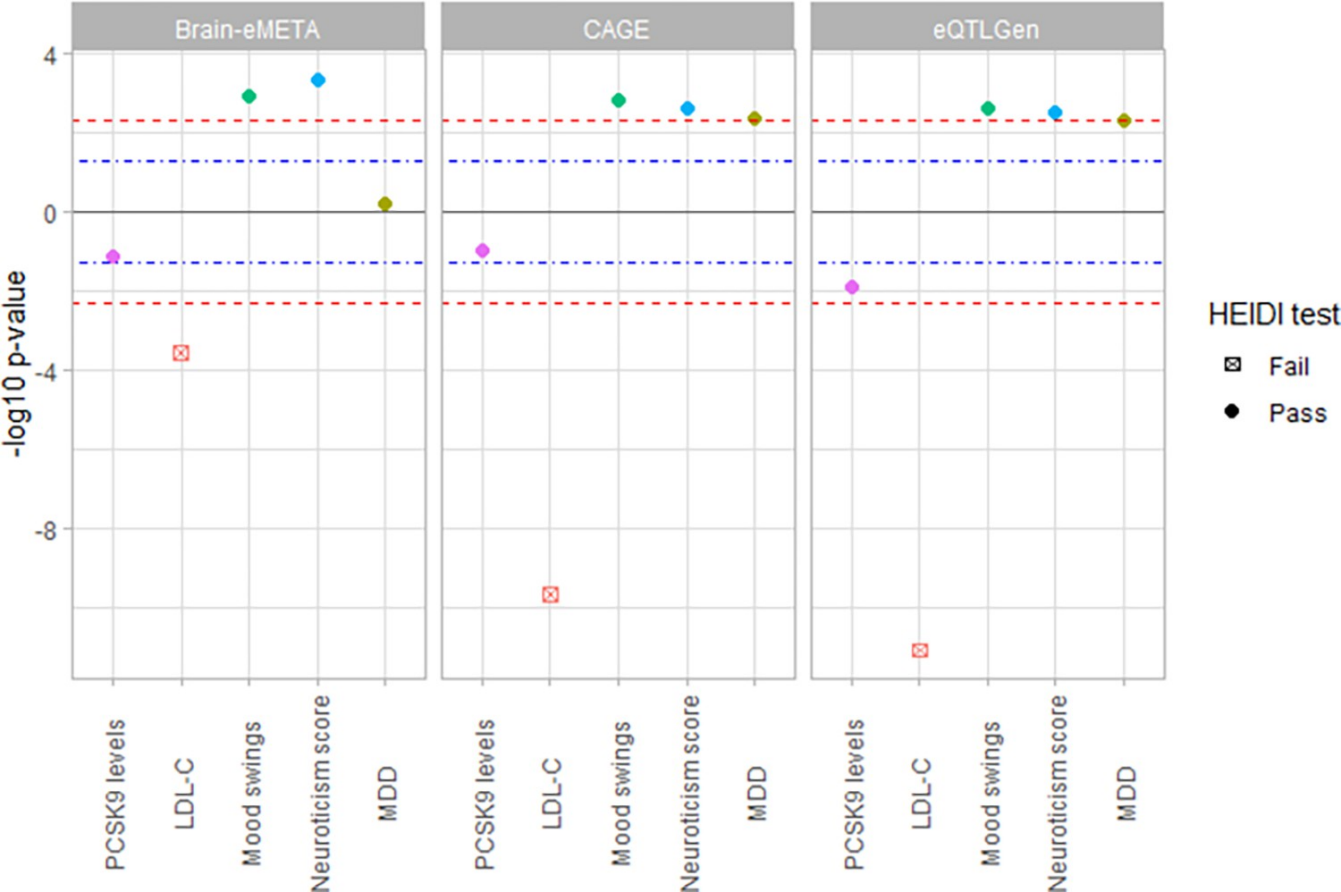
MR association of *USP24* gene-expression levels with phenotypes of interest in replication cohorts. The y-axis represents the -log_10_ p-value of the MR association, while the direction signifies the direction of effect of gene-expression reduction, which serves as a proxy to the corresponding drug effect. SMR multi-SNP analyses associations for CAGE consortium (peripheral blood), eQTLGen consortium (whole blood), and Brain-eMETA dataset (brain) for *USP24* gene-expression. The blue and red lines signify p-value of 0.05 and Bonferroni-corrected threshold for 5 traits per gene of 0.005.

Repeating the analyses in the eQTLGen consortium, CAGE consortium, and Brain-eMETA datasets, we found significant associations of *USP24* gene-expression with mood instability and neuroticism score for Brain-eMETA and CAGE consortium while there was suggestive significance for eQTLGen consortium. The direction of effect (reduced *USP24* gene-expression associated with reduced LDL-C but increased mood instability, and neuroticism) was consistent across all datasets. (Fig 3, S4 Table). GWAS summary statistic for significant *PCSK9* eQTLs was not available for these cohorts at the required thresholds.

### *USP24* might have implications on mood disorder traits

To assess whether eQTLs for *USP24* also demonstrated significant associations with neuroticism score and mood instability, which may indicate shared causal variants, we performed a colocalization analyses on the *USP24* locus. We found that the posterior probability for the H_4_ hypothesis (association with both trait 1 and trait 2 and they share the same causal variant) was greater than 0.8 for 19 tissues for mood instability (S7 Table), and 21 tissues for neuroticism score (S7 Table), indicating that for those tissues, the trait-association and the gene-expression association shared the same causal variants.

## Discussion

We investigated the potential effect of PCSK9i on mood disorder-related traits using MR, and publicly available GWAS and eQTL data. Genetically reduced *PCSK9* expression was significantly associated with reduced LDL-C and PCSK9 protein levels but not significantly associated with an increased risk of MDD, mood instability, or neuroticism scores. However, we demonstrated that genetically reduced expression of a neighbouring gene (± 1Mb), *USP24*, was significantly associated with an increased risk of mood instability, and neuroticism score.

The PCSK9 locus has only recently been implicated in GWAS, specifically in gene-based analyses of opioid use disorder [35]. The gene-based analyses use positional information to assign genes to variants, therefore it is possible that many of the variants would also be assigned to *USP24*. *USP24* is located approximately 175Mb downstream of *PCSK9* and many genetic variants are eQTLs for both genes. *USP24* encodes a ubiquitously expressed ubiquitin carboxyl-terminal hydrolase 24, a 2620-amino acid protein that belongs to ubiquitin-conjugating and deubiquitinating cysteine protease family whose main role is to clear abnormal proteins from cells and regulate cell survival [36, 37]. *USP24* gene-expression has low tissue specificity and is detected in all tissues with cytoplasmic protein expression in most cell types (Human Protein Atlas v21.0.proteinatlas.org) [38]. The DisGeNET knowledge platform’s analyses found *USP24* dysregulation to be associated with LDL-C levels (gene-disease score, GDA = 1), Parkinson disease (GDA 0.08), and various cancer types (GDA = 0.04–0.01) including lung cancer, multiple myeloma, T-cell lymphoma, and neuroblastoma [39]. The Locus-to-Gene (L2G) pipeline of Open Targets Genetics consortium finds *USP24* as a prioritised gene for GWAS variants associated with LDL-C (L2G score: 0.7), estimated glomerular filtration rate (L2G score: ~0.6), various CVD (L2G score: ~0.8), metabolic disorders (L2G score: ~0.01) and various cancers (L2G score: ~0.06) [40]. This is the first report of a role for *USP24* in mood instability, and neuroticism score, though it should be noted that mood instability is a component of the neuroticism score and could be driving the latter association. Understanding how *USP24* impacts mood traits requires further work. Investigating the impact of *USP24* on all of the traits/questions that contribute to neuroticism score would be an interesting starting point.

Strengths of our study included the use of cis-eQTLs that passed a strict association threshold in the discovery dataset, positive control outcomes (specifically circulating PCSK9 and LDL-C levels) and replication in 3 independent datasets (although Brain-eMETA overlaps with GTEx brain tissue data). We also used *LDLR* gene-expression level as another way to assess if PCSK9i were associated with mood-disorder traits via the main function of the drug, that being to increase LDLR levels. Moreover, it also showed that *USP24* gene-expression does not affect PCSK9 protein levels, suggesting that the effect of the PCSK9/*USP24* locus on mood-related traits is through a pathway independent of PCSK9. We also used all available tissues in GTEx for the analyses with the rationale that even when the disease specific tissues is known, *PCSK9* is expressed in multiple tissues and its non-LDLR specific effect in other tissues has not been proven to be null. A limitation of our analyses is the sample size of eQTL cohorts as they are far smaller than a conventional GWAS, and therefore may have provide limited confidence. Whilst replication in different cohorts and tissues has increased the confidence of our findings, GWAS summary statistic for the *PCSK9* eQTLs, or their proxies, that met the eQTL p-value threshold of 5x10^-8^ were unavailable in eQTLGen, CAGE, and Brain-eMETA consortium datasets. Moreover, as PCSK9’s main function is in the hepatic tissues, it would have been beneficial to analyse the effects in the liver. For example, Inclisarin, an small interfering RNA (siRNA) PCSK9i approved in 2021 by both the FDA and NICE, binds specifically to hepatocytes leading to a targeted reduction of PCSK9 secretion in the liver [41, 42], and hence analysing the effects in the hepatic tissues will be crucial. However, no significant eQTLs for *PCSK9* were present for the liver tissue in GTEx v8. This might be due to the lower sample size (n = 208) or technical reasons such as sample collection methods. In addition, we will also have to be mindful that cell types and environments of tissues used in both eQTL and GWAS studies may not be the same. For example, LDL-C GWAS conducted using DNA from blood samples collected from living population and clinical cohorts, while RNA estimates used for eQTL analyses in GTEx were from post-mortem tissues, leading to a potential variation in the regulatory environment of the tissue types.

Lastly, we have not focused on the magnitude of effect size (beta) as the reported effect sizes for the GTEx cohort is calculated in a normalised space and the magnitude of it cannot be used for any direct biological interpretation [43]. It is also noteworthy that in the GTEx cohort, there is a potential sample overlap for the calculation of eQTL summary statistics in different tissues as it is likely that the same individual was the donor for multiple tissues [19].

## Conclusion

Although our previous study demonstrated an association between the *PCSK9* locus and mood disorder-related traits [6], here we provide evidence that gene-expression modulation of *USP24* –and not *PCSK9 –*is responsible for the causal effects on mood instability, and neuroticism scores. PCSK9i are therefore unlikely to have an adverse impact on mood disorder-related traits, a reassuring finding for a drug class that is widening in use in many countries as costs levels start to decline.

## Supporting information

S1 Fig SMR association of *PCSK9* and *USP24* gene-expression levels with phenotypes of interest. The Y-axis represents the -log_10_ p-value of the MR association, while the direction signifies the direction of effect of gene-expression reduction, which serves as a proxy for PCSK9i. SMR multi-SNP analyses association of the available 25 tissues for *PCSK9* and *USP24* gene-expression in GTEx v8 for outcomes PCSK9 levels, LDL-C levels, and MDD. The blue and red lines signify p-value of 0.05 and Bonferroni-corrected threshold for 5 traits per gene of 0.005, respectively.
(TIF)

S2 Fig Pleiotropy assessment for PCSK9 eQTL and mood-disorder related trait using SMR. The Y-axis represents the -log_10_ p-value of the MR association, while the direction signifies the direction of effect of gene-expression reduction, which serves as a proxy for PCSK9i. SMR multi-SNP analyses association of the available tissues for expression of genes within 1Mb flanking of PCSK9 using only PCSK9 eQTL from GTEx v8 as IV for outcomes PCSK9 levels, LDL-C levels, mood swings, neuroticism score, and MDD. PCSK9 eQTLs having GWAS association with our outcomes are shared with *BSND* and *USP24*. The blue and red lines signify p-value of 0.05 and Bonferroni-corrected threshold for 5 traits per gene of 0.005, respectively.
(TIF)

S1 Table Details of GWAS used as outcomes. IEUGWAS id: study ID in the IEU GWAS database, Outcome trait: name of the GWAS trait, Cohort: name of the GWAS cohort, Sex: sex of the GWAS population, Population: ethnicity of the GWAS population, Unit: measurement unit of the GWAS trait, Sample size: sample size of the GWAS cohort, Ref (see manuscript): reference for the study. Please refer to the manuscript.
(XLSX)

S2 Table Power calculations. Cohort: study used for eQTL data, Tissue: eQTL summary statistics obtained from this tissue, n eQTL: sample size for eQTL specific to tissue, r2 eQTL: proportion of variance in exposure variable explained by SNPs in gene expression GWAS, Outcome: trait used as outcome for MR, Outcome category: trait category, βOLS: regression coefficient for the observational association between the exposure and continuous outcome variables, βyx: unknown true causal association between exposure and continuous outcome, σ2 eQTL: variance of the exposure variable, σ2 GWAS: variance of the continuous outcome variable, n GWAS: sample size of GWAS, n cases GWAS: sample size of cases for binary outcomes, True OR: true odds ratio of the outcome variable per standard deviation of the exposure variable, K: proportion of cases in binary outcome, Power: estimated power for the exposure-outcome combination.
(XLSX)

S3 Table SMR results for GTEx cohort. Cohort: cohort of the eQTL study, probeID: ID of the probe used in respective cohorts for gene of interest, ProbeChr: chromosome of the probe used in respective cohorts for gene of interest, Gene: gene analysed, Probe bp: base pair position of the probe bp used in respective cohorts for gene of interest, topSNP: eQTL/SNP with lowest p-value for gene-tissue combination, topSNP chr: chromosome of the topSNP, topSNP bp: base pair position of the topSNP, A1: effect allele of topSNP, A2: other allele of topSNP, Freq: allele frequency for A1, b GWAS: beta for topSNP in the GWAS study, se GWAS: SE for topSNP in the GWAS study, p GWAS: P-value for topSNP in the GWAS study, b eQTL: beta for topSNP in the eQTL cohort, se eQTL: SE for topSNP in the eQTL cohort, p eQTL: P-value for topSNP in the eQTL cohort, b SMR: beta for SMR analysis using the topSNP only, se SMR: SE for SMR analysis using the topSNP only, p SMR: P-value for SMR analysis using the topSNP only, p SMR multi: P-value for SMR analysis using all significant independent SNPs, p HEIDI: P-value for the HEIDI analysis. Blank if test wasn’t conducted, nsnp HEIDI: number of SNPs used for HEIDI analysis. Blank if less than 3 SNPs—analysis was not conducted, Tissue: tissue type used for eQTL summary statistics, Outcome: Traits analysed.
(XLSX)

S4 Table SMR results for replication cohorts. Cohort: cohort of the eQTL study, probeID: ID of the probe used in respective cohorts for gene of interest, ProbeChr: chromosome of the probe used in respective cohorts for gene of interest, Gene: gene analysed, Probe bp: base pair position of the probe bp used in respective cohorts for gene of interest, topSNP: eQTL/SNP with lowest p-value for gene-tissue combination, topSNP chr: chromosome of the topSNP, topSNP bp: base pair position of the topSNP, A1: effect allele of topSNP, A2: other allele of topSNP, Freq: allele frequency for A1, b GWAS: beta for topSNP in the GWAS study, se GWAS: SE for topSNP in the GWAS study, p GWAS: P-value for topSNP in the GWAS study, b eQTL: beta for topSNP in the eQTL cohort, se eQTL: SE for topSNP in the eQTL cohort, p eQTL: P-value for topSNP in the eQTL cohort, b SMR: beta for SMR analysis using the topSNP only, se SMR: SE for SMR analysis using the topSNP only, p SMR: P-value for SMR analysis using the topSNP only, p SMR multi: P-value for SMR analysis using all significant independent SNPs, p HEIDI: P-value for the HEIDI analysis. Blank if test wasn’t conducted, nsnp HEIDI: Number of SNPs used for HEIDI analysis. Blank if less than 3 SNPs—analysis was not conducted, Outcome: Traits analysed.
(XLSX)

S5 Table SMR results for *PCSK9* pleiotropic analyses. Cohort: cohort of the eQTL study, probeID: ID of the probe used in respective cohorts for gene of interest, ProbeChr: chromosome of the probe used in respective cohorts for gene of interest, Gene: gene analysed, Probe bp: base pair position of the probe bp used in respective cohorts for gene of interest, topSNP: eQTL/SNP with lowest p-value for gene-tissue combination, topSNP chr: chromosome of the topSNP, topSNP bp: base pair position of the topSNP, A1: effect allele of topSNP, A2: other allele of topSNP, Freq: allele frequency for A1, b GWAS: beta for topSNP in the GWAS study, se GWAS: SE for topSNP in the GWAS study, p GWAS: P-value for topSNP in the GWAS study, b eQTL: beta for topSNP in the eQTL cohort, se eQTL: SE for topSNP in the eQTL cohort, p eQTL: P-value for topSNP in the eQTL cohort, b SMR: beta for SMR analysis using the topSNP only, se SMR: SE for SMR analysis using the topSNP only, p SMR: P-value for SMR analysis using the topSNP only, p SMR multi: P-value for SMR analysis using all significant independent SNPs, p HEIDI: P-value for the HEIDI analysis. Blank if test wasn’t conducted, nsnp HEIDI: number of SNPs used for HEIDI analysis. Blank if less than 3 SNPs—analysis was not conducted, Tissue: Tissue type used for eQTL summary statistics, Outcome: Traits analysed.
(XLSX)

S6 Table Cross-tissue MR results. outcome: outcome analysed, exposure: exposure names (expression levels of the respective genes), method: MR methods used, nsnp: number of IV used for the MR method used, b: beta/effect size for the MR method used, se: Standard error for the MR method used, pval: P-value for the MR method used, Q: Cochrane Q value, Q df: Cochrane Q degrees of freedom, Q pval: Cochrane Q P-value, egger intercept: MR-Egger intercept value, se: MR-Egger standard error, pval: MR-Egger p-value.
(XLSX)

S7 Table Coloc results for USP24 and mood disorder traits. nsnps: number of SNPs used for coloc analyses, H0: posterior probabilities for colocalization—neither trait has a genetic association in the region, H1: posterior probabilities for colocalization -only gene-expression has a genetic association in the region, H2: posterior probabilities for colocalization -only GWAS trait has a genetic association in the region, H3: posterior probabilities for colocalization—both traits are associated, but with different causal variants, H4: posterior probabilities for colocalization—both traits are associated and share a single causal variant, tissue: GTEx tissue used, gwas_trait: trait in the GWAS used.
(XLSX)
