# Supplementary figures and images for: Investigating the potential impact of PCSK9-inhibitors on mood disorders using eQTL-based Mendelian randomization
Source: PLoS One. 2022 Dec 29;17(12):e0279381. doi: 10.1371/journal.pone.0279381 (PMC9799310; doi:10.1371/journal.pone.0279381)

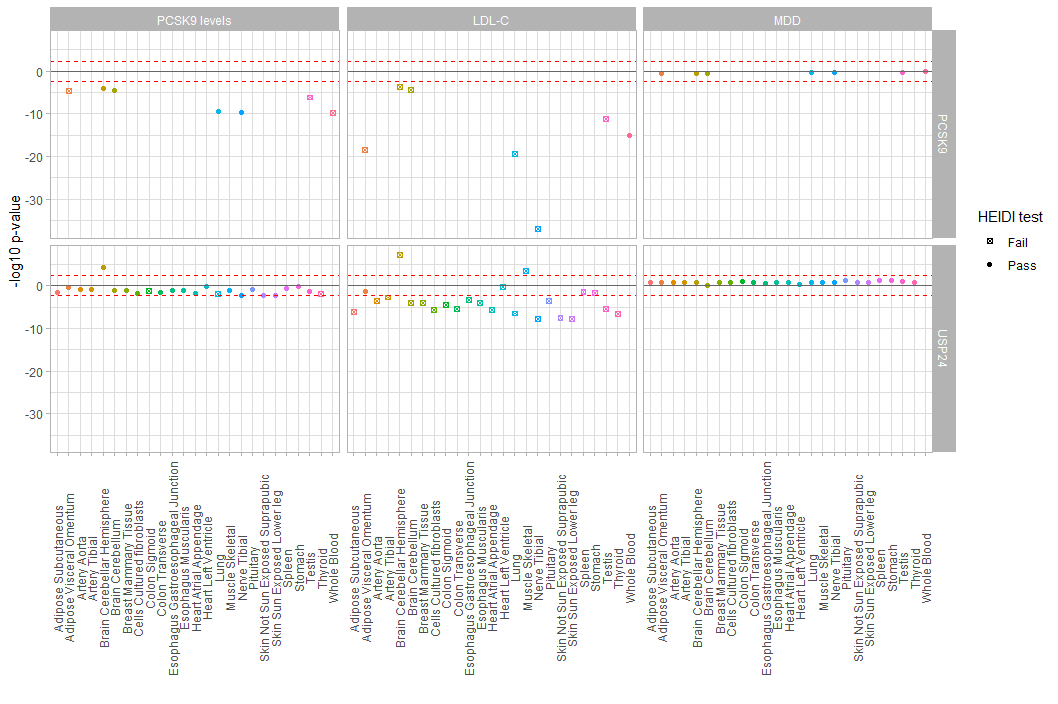

Supplement: S1 Fig — The Y-axis represents the -log10 p-value of the MR association, while the direction signifies the direction of effect of gene-expression reduction, which serves as a proxy for PCSK9i. SMR multi-SNP analyses association of the available 25 tissues for PCSK9 and USP24 gene-expression in GTEx v8 for outcomes PCSK9 levels, LDL-C levels, and MDD. The blue and red lines signify p-value of 0.05 and Bonferroni-corrected threshold for 5 traits per gene of 0.005, respectively. (TIF) [file pone.0279381.s001.tif]

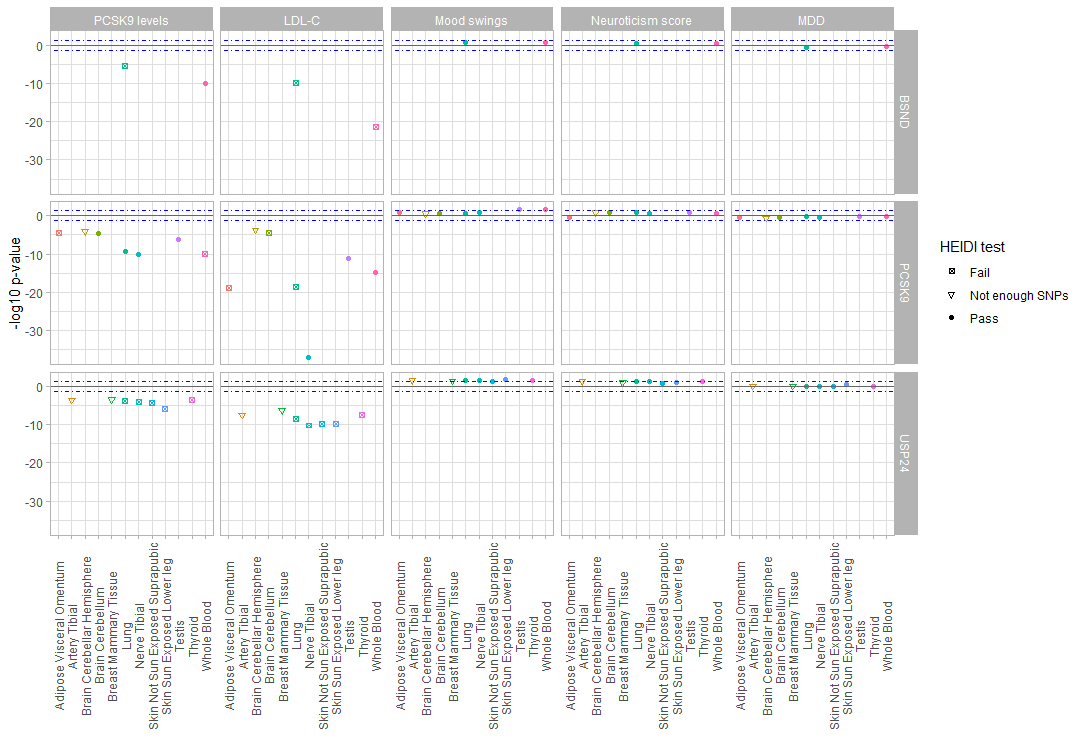

Supplement: S2 Fig — The Y-axis represents the -log10 p-value of the MR association, while the direction signifies the direction of effect of gene-expression reduction, which serves as a proxy for PCSK9i. SMR multi-SNP analyses association of the available tissues for expression of genes within 1Mb flanking of PCSK9 using only PCSK9 eQTL from GTEx v8 as IV for outcomes PCSK9 levels, LDL-C levels, mood swings, neuroticism score, and MDD. PCSK9 eQTLs having GWAS association with our outcomes are shared with BSND and USP24. The blue and red lines signify p-value of 0.05 and Bonferroni-corrected threshold for 5 traits per gene of 0.005, respectively. (TIF) [file pone.0279381.s002.tif]
